# Biochemical Factors Modulating Cellular Neurotoxicity of Methylmercury

**DOI:** 10.1155/2011/721987

**Published:** 2011-09-20

**Authors:** Parvinder Kaur, Michael Aschner, Tore Syversen

**Affiliations:** ^1^Department of Neuroscience, Norwegian University of Science and Technology, 7489 Trondheim, Norway; ^2^Departments of Pediatrics and Pharmacology and The Kennedy Center for Research on Human Development, Vanderbilt University Medical Center, B-3307 Medical Center North, 1162 21st Avenue, Nashville, TN 37232-2495, USA; ^3^Department of Neuroscience, Faculty of Medicine, Norwegian University of Science and Technology, Olav Kyrres Gate 3, 7489 Trondheim, Norway

## Abstract

Methylmercury (MeHg), an environmental toxicant primarily found in fish and seafood, poses a dilemma to both consumers and regulatory authorities, given the nutritional benefits of fish consumption versus the possible adverse neurological damage. Several studies have shown that MeHg toxicity is influenced by a number of biochemical factors, such as glutathione (GSH), fatty acids, vitamins, and essential elements, but the cellular mechanisms underlying these complex interactions have not yet been fully elucidated. The objective of this paper is to outline the cellular response to dietary nutrients, as well as to describe the neurotoxic exposures to MeHg. In order to determine the cellular mechanism(s) of toxicity, the effect of pretreatment with biochemical factors (e.g., N-acetyl cysteine, (NAC); diethyl maleate, (DEM); docosahexaenoic acid, (DHA); selenomethionine, SeM; Trolox) and MeHg treatment on intercellular antioxidant status, MeHg content, and other endpoints was evaluated. This paper emphasizes that the protection against oxidative stress offered by these biochemical factors is among one of the major mechanisms responsible for conferring neuroprotection. It is therefore critical to ascertain the cellular mechanisms associated with various dietary nutrients as well as to determine the potential effects of neurotoxic exposures for accurately assessing the risks and benefits associated with fish consumption.

## 1. Introduction

Methylmercury (MeHg) is a ubiquitous environmental toxicant [1]. Several catastrophic epidemics resulting from the consumption of food contaminated by MeHg have highlighted the potentially disastrous effects of MeHg on living organisms. Important examples include outbreaks in Minamata [2], Niigata [3], and Iraq [4]. MeHg is a potent neurotoxicant which affects both the developing and mature CNS [5, 6]. In infants, MeHg causes widespread and diffuse damage, whereas focal damage is caused in the adult brain. In adults, chronic MeHg poisoning results in the degeneration of the sensory cerebral cortex and the cerebellum, thereby leading to severe neurological disturbances, such as cerebellar ataxia and paresthesia, sensory and speech impairment, and the constriction of the visual field [4, 7, 8]. The pathological changes involve general neuronal degeneration with gliosis in the calcarine, and precentral and postcentral areas of the cerebral cortex, as well as the loss of granular cells in the cerebellar cortex [9]. In biological systems, MeHg exists only at a very low concentration as a free, unbound cation [10] which can bind to sulfhydryl groups (-SH) of amino acids with a very high affinity (log K in the order of 15–23) [10]. This affinity of Hg for sulphur and -SH groups is a major factor underlying the biochemical properties of MeHg, which, consequently, leads to its interference with the enzyme activities of several cellular targets. 

In the marine ecosphere, MeHg is sustained [11, 12] and, after bioaccumulation, is introduced into the human population through the dietary intake of fish and seafood products. [13–15]. MeHg toxicity due to the consumption of adulterated fish represents a major public health issue. Greater fish consumption in many cases is paralleled by increased MeHg intake [16]; however, conversely, lower maternal seafood intake has also been associated with higher risk for a suboptimal developmental outcome [17]. According to the Avon Longitudinal Study of Parents and Children (ALSPAC), the authors reported that maternal seafood intake during pregnancy of less than 340 g per week was associated with an increased likelihood for their children to fall into the lowest quartile for verbal intelligence quotient (IQ) when compared with mothers who consumed more than 340 g of seafood per week. Though Hg consumption was not assessed in this study, it is reasonable to assume that greater fish consumption was paralleled by increased MeHg intake. Moreover, several discrepancies in health outcomes in fish-eating populations have been reported, such as neurodevelopmental impairments in New Zealand [18–20] and the Faroe Islands [21, 22], as opposed to the beneficial effects noted in Canada [23], the Seychelles [24, 25], Peru [26], and the United States [17, 27–30]. Additionally, laboratory studies have also shown that dietary factors, such as selenium, cysteine, protein, fat, fiber, and vitamin contents can modulate the toxicity and excretion of mercury [31, 32]. A previous study [33] has also shown a significantly higher rate of fecal excretion as well as a lower degree of MeHg accumulation in the brains of rats fed naturally contaminated fish as compared to rats fed fish containing chemically added MeHg. The above-mentioned studies indicate that, in addition to intrinsic, genetic factors, the phenotypic responses to MeHg exposure may ultimately depend on a number of complex interactions within biological systems involving both mercury and various dietary factors. It is therefore important to study the effect(s) of confounding dietary factors that occur when fish is consumed on MeHg distribution and neurotoxicity. In this respect, it must be noted that different types of fish accumulate different concentrations of nutrients and contaminants [34–36]. Therefore it is of considerable interest to determine how each component acts individually (as well as with others) and influences the potential risk from MeHg exposure. These cellular and molecular mechanisms of MeHg action, as well as the underlying processes of its interaction with dietary components have yet to be defined, especially in specific central nervous system (CNS) targets. Accordingly, this paper focuses on studies directed toward estimating the effect(s) of dietary modifiers on MeHg neurotoxicity, potentially providing information about critical cellular mechanisms responsible for conferring neuroprotection from a diet that includes MeHg-contaminated fish.

## 2. MeHg-Induced Oxidative Effects: Reactive Oxygen Species (ROS) Generation and Glutathione (GSH) Depletion

The disruption of redox cellular homeostasis by an excess of ROS formation, which leads to cumulative oxidative stress appears to play a key role in the *in vivo* pathological process of MeHg intoxication [37–42]. Conversely, several studies have demonstrated the partial amelioration of MeHg toxicity in the presence of antioxidants by the inhibition of ROS generation [40, 43, 44]. Although the critical role of oxidative stress in the pathogenesis of MeHg cytotoxicity has been clarified, the molecular mechanisms underlying MeHg-mediated oxidative stress have not yet been fully elucidated. A major source of MeHg-induced increases in ROS generation may be the mitochondrial electron transport chain. The damaged mitochondrion increases oxidative stress, leading to a decrease in defense mechanisms, such as reduced GSH content, which represents one of the principal endogenous antioxidants. In addition, binding to GSH is reported to be responsible for the excretion of MeHg. Therefore, decreased GSH levels usually parallel increased oxidative stress due to MeHg exposure [45–49]. However, two epidemiological studies associating oxidative stress and MeHg exposure [50, 51] have shown both an increase and a decrease in GSH levels with increased total Hg levels. This suggests that MeHg can increase ROS which may either inhibit GSH levels or initiate an adaptive response to oxidative stress by increasing GSH levels. Moreover, studies of human populations, although of direct interest, cannot be controlled for multiple confounding variables. This obstacle can be overcome by conducting studies on laboratory animals; such investigations can identify the mechanisms of action by which neurotoxicants and neuroprotectants interact.

## 3. Role of GSH Modulators on MeHg-Induced Neurotoxicity

Upregulation [52], or the induction of an increased synthesis of GSH [45], has been reported to provide neuroprotection against MeHg-induced neurotoxicity. A similar alleviation in MeHg-induced cytotoxicity and oxidative stress has been reported with N-acetyl cysteine (NAC) supplementation [39, 53–55]. The mechanisms involved in protection afforded by NAC include increased intracellular GSH [54, 55] as well as a transient increase in the urinary excretion of MeHg, which was shown to cause a decrease in the level of MeHg in both the adult brain and the fetus [53, 56]. In addition, the increased amount of GSH in cortical, as compared to cerebellar, astrocytes has been reported to account for the increased MeHg-induced ROS production in cerebellar astrocytes [55].

Conversely, the depletion of intracellular GSH with diethyl maleate (DEM) has been reported to increase cell-associated MeHg and MeHg-induced ROS [48, 54, 55]. The underlying mechanism of this process involves the conjugation of free sulfhydryl groups of GSH with DEM, which results in the distinct depletion of GSH. Also, gestational exposure to MeHg has been reported to cause the dose-dependent inhibition of cerebral GSH levels, an outcome which could be correlated with increased lipid peroxidation in the pup brain [57]. These biochemical alterations were found to endure even after Hg tissue levels decreased, thus indicating permanent functional deficits observed after prenatal MeHg exposure as well as an additional molecular mechanism by which MeHg induces prooxidative damage in the developing CNS.

In summary, changes in intracellular MeHg content with GSH modulation provide an explanation for the increased susceptibility of certain cell types towards MeHg-induced oxidative stress [54, 55].

## 4. Role of DHA in Modulating MeHg-Induced Neurotoxicity

DHA *cis*-4,7,10,13,16,19-docosahexaenoic acid, is one of the most abundant polyunsaturated fatty acids (PUFA) in the phospholipid fractions of the mammalian brain [58, 59]. Both seafood and breast milk serve as major dietary routes of MeHg [60, 61] and DHA [62–65]. The ability of DHA to affect ROS is controversial, as several contrasting studies have documented the ability of DHA to decrease the level of lipid peroxide [66–68] and to cause free-radical-mediated peroxidation in the brain [69–71]. DHA have been reported to modulate MeHg toxicity [33, 72–74]. These studies have demonstrated the beneficial effects of DHA on using a DHA-enriched diet against MeHg-induced decreases in serum albumin levels, changes in mitochondrial membrane potential, and developmental defects. However, other contradictory studies have reported no protection against MeHg-induced behavioral defects [75, 76]. It is therefore important to identify the biochemical mechanisms involved in the DHA protection against MeHg neurotoxicity. 

Kaur and colleagues [77, 78] demonstrated that pretreatment with DHA was associated with reduced cell-associated MeHg in neuronal cell lines and primary cells. In addition, decreased ROS and unchanged GSH levels were found in primary cultures, whereas increased ROS and GSH depletion were found in C6 cells [77, 78]. These differences with respect to the effect of DHA on oxidative stress could be due to the fact that the growth of cancerous cells is inhibited by DHA as compared to noncancerous cell types [71, 79]. Indeed, another recent study has shown that fish oil offers significant DNA protection as well as anti-inflammatory effects in the absence of changes in GSH levels [50]. These observations strongly suggest that DHA may neuroprotect against MeHg-induced ROS generation even in the absence of significant changes in GSH levels.

## 5. Role of Selenomethionine in Modulating MeHg-Induced Neurotoxicity

Selenium (Se) is an essential trace element known to accumulate in significant amounts in numerous species of seafood [80, 81]. The majority of Se in fish is in the organic form, selenomethionine (SeM) [82, 83], and is more bioavailable than are inorganic forms [84]. Selenium has also been detected in human milk [85]. The modulating effect of Se on MeHg toxicity was discovered when researchers observed that marine mammals could accumulate exceptionally high concentrations of Hg and Se compounds without displaying obvious symptoms of intoxication [86, 87]. Several subsequent studies later confirmed that the toxic effects of both organic and inorganic Hg were prevented by Se compounds [88–92]. Treatment with different Se compounds has been shown to effectively protect cells against different toxic effects induced by MeHg exposure, such as cytotoxicity, fetotoxicity, neurotoxicity, and developmental and neurobehavioral toxicity [93–97]. In addition, Se deficiency has been shown to potentiate the adverse effects of MeHg toxicity in rodents [98, 99]. 

With regard to epidemiological studies and Se content, it is important to note that Faroe Islanders, by virtue of a whale meat diet, are generally exposed to MeHg levels that are in excess of Se levels [100], whereas the Seychellois are largely ocean fish consumers, and Se molar concentrations tend to greatly exceed MeHg concentrations in this seafood source [101]. In addition, the dietary Se status in the New Zealand population was extremely poor at the time of the study [102]. This distinction could be one explanation for the different effects noted in these studies, although additional evidence is needed to support this hypothesis [103]. Therefore, developing a better understanding of the mechanisms associated with the interaction of MeHg and Se is of particular necessity.

Several studies have indicated that the mechanism underlying Se's ability to ameliorate MeHg toxicity is related to an antioxidant effect [104–108], which includes the formation of GSH [109], higher glutathione peroxidase (GPx) activity [85], increased selenoprotein levels [110–112], and the reduction of organic hydroperoxides [113–115]. Additionally, studies have shown that binding of MeHg [116, 117] and the formation of a highly stable organic MeHg-selenocysteine complex [98] also influence the accumulation of MeHg in tissues [118–121] and the uptake of MeHg in cells [114, 122–124]. Furthermore, Se is known to enhance the excretion of MeHg [56, 125], and a recent study has shown [126] that SeM can demethylate MeHg under physiologically and environmentally relevant conditions. Hence, the interactive effects between MeHg and SeM result in reduced cell-associated MeHg and prooxidant response from MeHg.

## 6. Role of Trolox in Modulating MeHg-Induced Neurotoxicity

Seafood serves as a source of vitamins, with estimates ranging between 4.84 and 17.90 *μ*g vitamin E per gm of fish [127], which makes this vitamin the most significant physiologic membrane-associated antioxidant available from seafood. Trolox (6-hydroxy-2,5,7,8-tetramethylchroman-2-carboxylic acid), a water soluble analog of vitamin E [128], serves as a better antioxidant than vitamin E [129, 130] due to its improved access to the hydrophilic compartments of the cells [131], as well as its stoichiometric properties [132]. Trolox scavenges free radicals [59, 133–137] via the H-donating groups [128, 138]. Treatment with Trolox has been reported to protect against MeHg-induced cytotoxicity [139], the decrease in mitochondrial electron transport system enzyme activities, and the increase of mRNAs of antioxidant enzymes [108, 140]. Trolox treatment has also been shown to reverse ROS induction by MeHg in primary astrocyte cultures [44] and to prevent MeHg-induced oxidative stress [141], where the modulating effect of Trolox on cellular ROS levels was not accompanied by changes in cellular MeHg, GSH, or MTT activity [141]. These findings indicate that Trolox affords protection against ROS by the direct quenching of free radicals and not by MeHg chelation or by the induction of increased levels of GSH or mitochondrial enzymes. In fact, it has been previously shown that *in vivo* protection with Trolox does not affect intracellular GSH [142, 143] or MeHg levels [140]. The recognition of the protective effects of Trolox and the identification of its mechanisms via *in vitro* models establish that vitamin-dependent antioxidant defences are important factors in specific cells for attenuating the neurotoxic effects of a MeHg-contaminated fish diet.

## 7. Discussion

Fish is not only an excellent nutritional source of protein, vitamins, zinc, and other minerals, but it is also a source of exposure to MeHg [144, 145]. One of the leading controversies in the MeHg literature originates from advisories concerning the consumption of fish [146] and from uncertainties in documentation from various regulatory agencies regarding the effects of MeHg. The Joint FAO/WHO Expert Committee on Food Additives reported in 1978 that “*the fetus may be more susceptible to MeHg toxicity than the adult*” [147]. The United States White House in 1998 convened an international workshop where a variety of possible uncertainties and confounders important to MeHg toxicity evaluation were discussed. Their conclusions stated, “*Even when dietary stresses and co-exposures to other chemicals could plausibly enhance or alter risk, it was still deemed that there are inadequate data on this subject to draw meaningful conclusions at this time*” [148]. Later, in 2000, the National Academy of Sciences committee reported that, “*60,000 children in the United States were at risk as a result of prenatal exposure”* [149]. However, no justification or explanation for that conclusion was provided [16, 150]. The issue that poses a significant dilemma for both consumers and regulatory authorities is whether fish consumption should be encouraged for its nutritional benefits to the developing brain or, conversely, whether fish consumption should be discouraged due to the possible adverse effects of MeHg on the developing CNS. This nutrition versus neurotoxicity controversy can be addressed by estimating the effects of dietary factors on MeHg-induced toxicity as well as by determining the mechanisms behind such effects. A thorough assessment of coexposure from dietary nutrients as well as neurotoxic exposures would offer valuable information for accurately determining the risks and benefits of fish consumption [151, 152].

**Figure 1:**
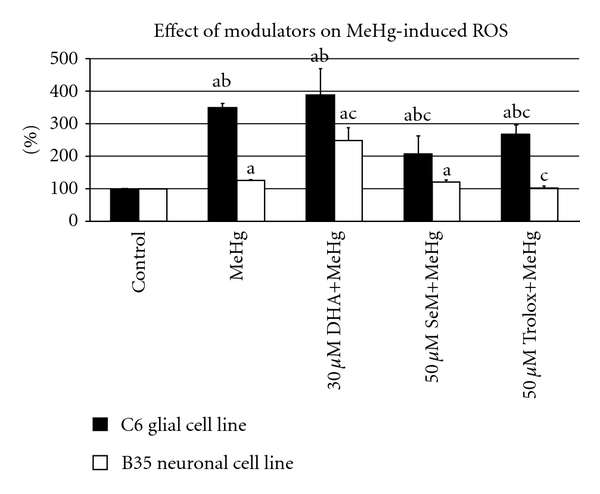
Effect of different modulators on MeHg-induced cellular ROS in C6-glial and B35-neuronal cell lines. Results are expressed as mean ± standard deviation (*n* = 8 replicates for each cell type in two independent experiments). Superscript (a) indicates *P *< 0.05 for control versus each type of treatment; (b) indicates *P *< 0.05 for C6 versus B35 cell line for each type of treatment; (c) indicates MeHg versus DHA/SeM or Trolox+MeHg-treated group. Values represented the percentage of activity relative to control cells.

This paper explores the mechanisms associated with MeHg and dietary nutrients obtained from the consumption of seafood. The toxicity of MeHg has been reported to be caused by a reduction in the amount of intracellular GSH [45, 46, 48], which leads to the augmentation of ROS formation [37, 40–44, 153]. This paper investigates the effects of MeHg on oxidative stress and details the role played by GSH in modifying these effects. It also identifies the biochemical mechanisms underlying exposure to GSH, DHA, Se, Trolox, and MeHg, where these modifiers have been shown to effectively decrease MeHg-induced ROS (Figure 1). In addition, it is important to note that the interaction between these dietary nutrients may have an effect on overall toxicity. For example, the benefits from Se against MeHg toxicity can be influenced by the intake of long-chain, polyunsaturated fatty acids (LCPUFAs) [65, 154]. It has also been shown that the shape of the dose-effect curve for Hg is dependent upon the co-exposure of dietary components such as Se and vitamin E [145]. This paper, concludes that GSH, DHA, Se and Trolox are strong confounders in the association of MeHg toxicity and that the interaction between them may affect the cellular oxidative status. Thus, it is necessary to consider different confounders and the various mechanisms by which they interact with Hg when investigating the potential beneficial effects of fish consumption. Indeed, doing so would provide valuable insight for developing a better understanding of the benefits and risks of fish consumption, acknowledging both the proven beneficial nutrients as well as the potentially dangerous contaminants contained in this important food source. Furthermore, such information would also assist public health authorities as they seek to advise the populace and as they undertake efforts to formulate appropriate dietary recommendations for consumers of fish and seafood products.
